# The First Nations Food, Nutrition and Environment Study (2008–2018)—rationale, design, methods and lessons learned

**DOI:** 10.17269/s41997-021-00480-0

**Published:** 2021-06-28

**Authors:** Hing Man Chan, Karen Fediuk, Malek Batal, Tonio Sadik, Constantine Tikhonov, Amy Ing, Lynn Barwin

**Affiliations:** 1 Department of Biology, University of Ottawa, 30 Marie Curie, Ottawa, ON K1N 6N5 Canada; 2 Département de nutrition, Faculté de Médecine, Pavillon Liliane de Stewart, Université de Montréal, CP 6128 succ. Centre-Ville, Montréal, QC H3T 1A8 Canada; 3 Centre de recherche en santé publique de l’Université de Montréal et du CIUSS du Centre-sud-de-l’Île-de-Montréal (CReSP), 7101 avenue du Parc, Montréal, HQ H3N 1X7 Canada; 4 Assembly of First Nations, 55 Metcalfe Street, Suite 1600, Ottawa, ON K1P 6L5 Canada; 5 First Nations and Inuit Health Branch, Department of Indigenous Services Canada, Ottawa, Canada

**Keywords:** Indigenous, First Nations, Food safety, Food security, Nutrition, Participatory research, Autochtones, Premières nations, salubrité des aliments, sécurité alimentaire, nutrition, recherche participative

## Abstract

**Objective:**

To describe the rationale, the participatory nature of the methodology, and the lessons learned during the First Nations Food, Nutrition and Environment Study (FNFNES), a community-based participatory research project implemented in eight Assembly of First Nations regions, which includes the entirety of Canada south of the 60^th^ parallel.

**Methods:**

FNFNES respected the First Nations principles of Ownership, Control, Access and Possession (OCAP®) (https://fnigc.ca/ocap). A random sampling strategy based on an ecosystem framework comprising 11 ecozones was adopted to collect representative nutritional and environmental health results for all First Nations adults living on-reserve south of the 60^th^ parallel. Data collection occurred during the fall months from 2008 to 2016. Respective First Nations were involved in the planning and implementation of data collection for the five principal components: household interviews, tap water sampling for metals, surface water sampling for pharmaceuticals, hair sampling for mercury, and traditional food sampling for contaminants.

**Results:**

A total of 6487 adults from 92 First Nations participated in the Study (participation rate 78%). A higher percentage of females (66%) participated than males (34%). The average age of males and females was similar (44 and 45 years, respectively). This study offers a novel body of coherent and regionally representative evidence on the human dimension of the ongoing environmental degradation affecting First Nations.

**Conclusion:**

FNFNES serves as a good example of participatory research. We encourage public health professionals to develop policy and programs building on the participatory dimension of the research as well as on its results. The information collected by the FNFNES is also important for community empowerment, environmental stewardship and the general promotion of good health by and for First Nations peoples in Canada.

## Résumé

**Objectifs:**

Décrire la justification, la nature participative de la méthodologie et les leçons apprises dans le cadre de l’Étude sur l’alimentation, la nutrition et l’environnement des Premières Nations (EANEPN), un projet de recherche participative communautaire mis en œuvre dans huit régions de l’Assemblée des Premières Nations, qui comprend l’ensemble du Canada au sud du 60^e^ parallèle.

**Méthodes:**

L’EANEPN a respecté les principes de propriété, de contrôle, d’accès et de possession des Premières Nations (PCAP®) (https://fnigc.ca/ocap). Une stratégie d’échantillonnage aléatoire basée sur un cadre écosystémique composé de 11 écozones a été adoptée pour recueillir des résultats représentatifs de la santé nutritionnelle et environnementale pour tous les adultes des Premières Nations vivant dans les réserves au sud du 60^e^ parallèle. La collecte de données a eu lieu au cours des mois d’automne de 2008 à 2016. Les Premières Nations respectives ont participé à la planification et à la mise en œuvre de la collecte de données pour les cinq principales composantes : entrevues avec les ménages, échantillonnage de l’eau du robinet pour les métaux, échantillonnage de l’eau de surface pour les produits pharmaceutiques, échantillonnage des cheveux pour le mercure, et échantillonnage des aliments traditionaux pour les contaminants.

**Résultats:**

Au total, 6 487 adultes de 92 Premières Nations ont participé à l’étude (taux de participation de 78 %). Un pourcentage plus élevé de femmes (66 %) a participé que d’hommes (34 %). L’âge moyen des hommes et des femmes était similaire (44 et 45 ans, respectivement). Cette étude offre pour la première fois un ensemble de preuves cohérentes et représentatives à l’échelle régionale sur la dimension humaine de la dégradation continue de l’environnement affectant les Premières Nations.

**Conclusion:**

L’EANEPN est un bon exemple de recherche participative. Nous encourageons les professionnels de la santé publique à élaborer des politiques et des programmes en s’appuyant sur la dimension participative de la recherche ainsi que sur ses résultats. Les informations recueillies par L’EANEPN sont également importantes pour l’autonomisation des communautés, la gestion de l’environnement et la promotion générale de la santé par et pour les peuples des Premières Nations au Canada.

## Introduction

“First Nations” refers to one of three distinct groups recognized as “Indigenous” in Canada. There are 634 First Nations communities (also known as reserves) in Canada, with over 50 distinct nations and language groups across the country (Assembly of First Nations 2020). There were 509,016 individuals with status under the Indian Act living on-reserve in 2017 (Government of Canada 2020). There remain large gaps in overall health between First Nations and the non-Indigenous population (NCCAH 2013). For example, 56% of First Nations reported being diagnosed with one or more chronic conditions, compared with 48% of the non-Indigenous population (Statistics Canada 2013). The health and well-being of individuals and communities are determined by a broad range of factors, including the social determinants of health, diet and lifestyle, genetics and the state of the environment. The social determinants of health play a key role in health inequities: those who have more advantages tend to have better health (Frohlich et al. 2006; Mikkonen and Raphael 2010). For millennia, First Nations have relied on locally adapted traditional food systems and diverse resource management and food production technologies (Deur and Turner 2005; Waldram et al. 1995). However, there has been a gradual dietary transition away from traditional food due to a multitude of factors, including a decline in the availability, quality and safety of, as well as access to, traditional food due to industrialization, pollution, climate change, government regulations, financial and time constraints, and cultural losses due to the legacy of the residential school system (Kuhnlein et al. 2013; Kuhnlein and Receveur 1996; Turner et al. 2013). Traditional food has key nutritional, cultural, spiritual and economic values for First Nations peoples and is often more nutrient-dense than commercially available “market” or store-bought food replacements (Batal et al. 2018; Batal et al. 2017). The decline of traditional food use increases the risk of malnutrition and induces a rise in nutrition-related health problems such as anemia, heart disease, obesity, osteoporosis, cancer, infections, diabetes and tooth decay (Kuhnlein and Receveur 1996). The health and nutrition of First Nations peoples can be strongly related to social disparities, the external erosion of traditional lifestyles, high food insecurity and poor quality diets (Adelson 2005; Batal and Decelles 2019; Kuhnlein and Receveur 1996; Power 2008; Willows et al. 2011; Willows 2005). Increasing industrialization in the last century has led to varying degrees of pollution in all ecosystems. Many health problems (e.g., cancer, diabetes, low infant weight) may be related to the presence of chemical contaminants in the environment (Hectors et al. 2011; Lee et al. 2011; Li et al. 2006; Institute of Medicine 2007). Therefore, it is critical to increase knowledge on the nutritional and environmental health status of First Nations.

Over the past 50 years, the Government of Canada has conducted three National Nutrition surveys (1970–1972 Nutrition Canada National Survey [NCNS], 2004 and 2015 Canadian Community Health Survey [CCHS]-Nutrition) and six Total Diet Studies (TDS) to understand the eating patterns, diet quality and environmental safety of store-bought foods for the general population. These studies, however, have been of limited value to First Nations. First Nations living on-reserve were not included in the 2004 and 2015 CCHS-Nutrition surveys (Statistics Canada 2017), and only store-bought foods have been examined in the TDS (Dabeka and Cao 2013). The 1970–1972 NCNS included 29 First Nations (27 First Nations south of the 60^th^ parallel and two First Nations in the Northwest Territories). However, the participation rate was 30%, and only one report was published containing aggregated nutrient intake results without food quality and consumption patterns (Health Canada 1975). Two decades later, fish and game consumption estimates, combined for First Nations and Inuit, were derived from unpublished anonymized 24-hour recalls from the NCNS with no distinction by geographic region or cultural identity (Richardson 1997). Nevertheless, these estimates have been recommended as a standard proxy for human health risk assessment when no local data exist (Health Canada 2010). Therefore, there is a critical data gap in the regional-specific diet composition, particularly the variety and amount of traditional food harvested locally, of First Nations living on-reserve.

First Nations in different geographical areas face unique environmental problems due to the nature and levels of environmental pollution and the degree to which their diets are obtained from local environments. Prior to the present study, the only comprehensive regional-level dietary data available for First Nations were from dietary studies conducted in the 1990s in the Yukon and Northwest Territories (Kuhnlein et al. 2001; Batal et al. 2005; Donaldson et al. 2010; Laird et al. 2013; Crown-Indigenous Relations and Northern Affairs Canada [CIRNAC] 2018). Therefore, it is important to obtain representative data on the nutritional composition and contaminant concentrations in traditional food commonly used by First Nations in the areas studied.

The goal of the First Nations Food, Nutrition and Environment Study (FNFNES, hereafter the “Study﻿”) was to obtain representative baseline data on food use patterns and exposure to contaminants to provide the information needed for the promotion of healthy environments and healthy food for healthy First Nations. The primary objectives were to (1) determine consumption patterns of traditional and market foods on-reserve; (2) collect traditional food and drinking water to determine dietary exposure to environmental contaminants; (3) determine the nutritional value of the mixed diet; and (4) document food security. A draft report presenting the descriptive results was released (Chan et al. 2019) primarily for the participating First Nations to respect the commitment that results be reported back to the First Nations before being released to the public. This paper presents the details of the rationale, study design, and general methods and a summary of participation results for FNFNES for the scientific community. We aim to describe the participatory nature of the Study, with emphasis on the importance of being respectful and engaging with local First Nations so they can utilize the information generated to improve their communities’ health, nutrition and environment. The lessons learned from the Study and the significance of the results are discussed. Detailed methods and findings for each component are presented in the accompanying papers in this Special Issue of the *Canadian Journal of Public Health* (Batal et al. 2021a–e; Marushka et al. 2021a, 2021b; Chan et al. 2021; Tikhonov et al. 2021; and Schwartz et al. 2021a, 2021b).

## Methods

### Participatory approach

From the start, the FNFNES recognized that First Nations needed to have an equal and participatory role at all levels of the research. Overarching First Nations support for the Study was received through a resolution passed by the Chiefs-in-Assembly in 2007 at the Assembly of First Nations (AFN) Annual General Assembly, and the AFN participated as one of the principal investigating partners throughout the Study. At the regional level, before implementation, First Nations provincial/territorial organizations were consulted. Regional Chiefs approved the Study and provided guidance on approaches to address the needs of specific local environmental issues or concerns and logistics needs in their respective regions. Such information has helped the Study ensure the best “snapshot” of regional representation at the time of data collection.

First Nations that were randomly selected to participate were initially contacted by the AFN and invited to attend a 2-day preparatory methodology workshop held in a central location in each region. At the respective workshops, representatives from the communities were introduced to the project, and they discussed the research objectives, methodology and implementation. They were consulted about the traditional foods that are consumed in the region and the appropriateness of the questionnaire. These workshops were an important opportunity for First Nations to provide input into the methodology and suggest changes to ensure that the Study would meet their needs. FNFNES was then introduced to leadership and the wider community by the research team in community meetings. The First Nations were asked to confirm their participation and to sign a Community Research Agreement, Funding Transfer Agreement and Band Council Resolution (if the First Nation deemed it to be required). The Community Research Agreement was presented as a draft so that the First Nation could make changes to the wording as it saw fit. Each First Nation was responsible for its data collection and the administration of funds, while the funding itself, support and training were provided by the FNFNES. The signing of the agreement marked the formal beginning of research activities.

### Study design

FNFNES used a single approach, with largely identical tools and methodology to conduct a regional-level survey of First Nations adults living on-reserve in the eight AFN regions located south of the 60^th^ parallel in Canada (British Columbia, Alberta, Saskatchewan, Manitoba, Ontario, Quebec-Labrador, and Atlantic (New Brunswick, Newfoundland, Nova Scotia and Prince Edward Island)) (Chan et al. 2019). To ensure that the diversity of diets of First Nations was represented, a random sampling strategy of communities was adopted, based on an ecosystem framework that included 11 ecozones, i.e., regions/areas identified based on the distribution patterns of plants, animals, geographical characteristics and climate. Therefore, regional results can represent the region as a whole, even though not all First Nations participated. Data collection occurred from 2008 to 2016 during the fall months (September to mid-December) in each region to ensure the comparability of results.

### Sampling strategy

Statistics Canada provided support for the sampling methodology and random sample selection.

The Study’s goal was to survey 100 adults from 100 First Nations in eight AFN regions. The final sampling framework was created with an allocation of 92 randomly selected First Nations: the number of communities allocated to each region was proportional to the square root of the number of communities within it that had a population on-reserve at the time of the initial sampling. The survey design also allowed for eight purposely selected First Nations. These First Nations were selected due to contamination concerns as well as availability of previously published data, and to enhance cultural and ecosystem diversity. Since these First Nations were not randomly selected, their results were not weighted when regional results were tabulated. In each AFN region, First Nations were further stratified into ecozones to ensure the diversity of diets was represented. The sample was proportionally allocated between the ecozone strata, except in ecozones with a very small number of communities, in which case all the communities were chosen. The selection of communities was made independently for each stratum. Within each selected community, a random sampling of 125 households was undertaken. A larger sample of households than the desired 100 was selected to adjust for expected refusal/non-response (25%). For communities with fewer than 125 households, every household in the community was selected. At the household level, a random selection of one adult took place (if there was more than one eligible adult, the research assistant was asked to select the person living in the household whose birthday was next). Participants had to be 19 years of age and older, be able to provide written informed consent, and self-identify as a First Nations person living on the reserve.

A total of 117 communities were approached to participate in the Study; 82 were randomly selected, nine were pre-selected with certainty either due to population size or if they were the sole community within an ecozone, and eight were purposely selected. Where communities chose not to participate, replacement communities within the same ecozone were approached. For logistical reasons and due to the larger number of selected First Nations, data collection took place over two years in the regions of British Columbia and Ontario.

### Weighting adjustment

Estimation weights were calculated to ensure that the data reflected the whole population from which they were drawn for each region. The data were weighted to adjust for non-response at three levels: communities, households and individuals. Since the FNFNES data were collected over a period of several years, an adjustment factor was created to account for population changes between 2008 and 2017 for the preparation of the summary statistics. A ratio of populations was calculated by dividing the 2017 population by the reference year population used in the weighting estimate documents for a particular AFN region. Adjustment factors were calculated individually for each First Nation and applied to the 501 weight variables of each FNFNES record (the estimation weight and the 500 replication or bootstrap weights) for that community.

### Principal study components

**Fig. 1:**
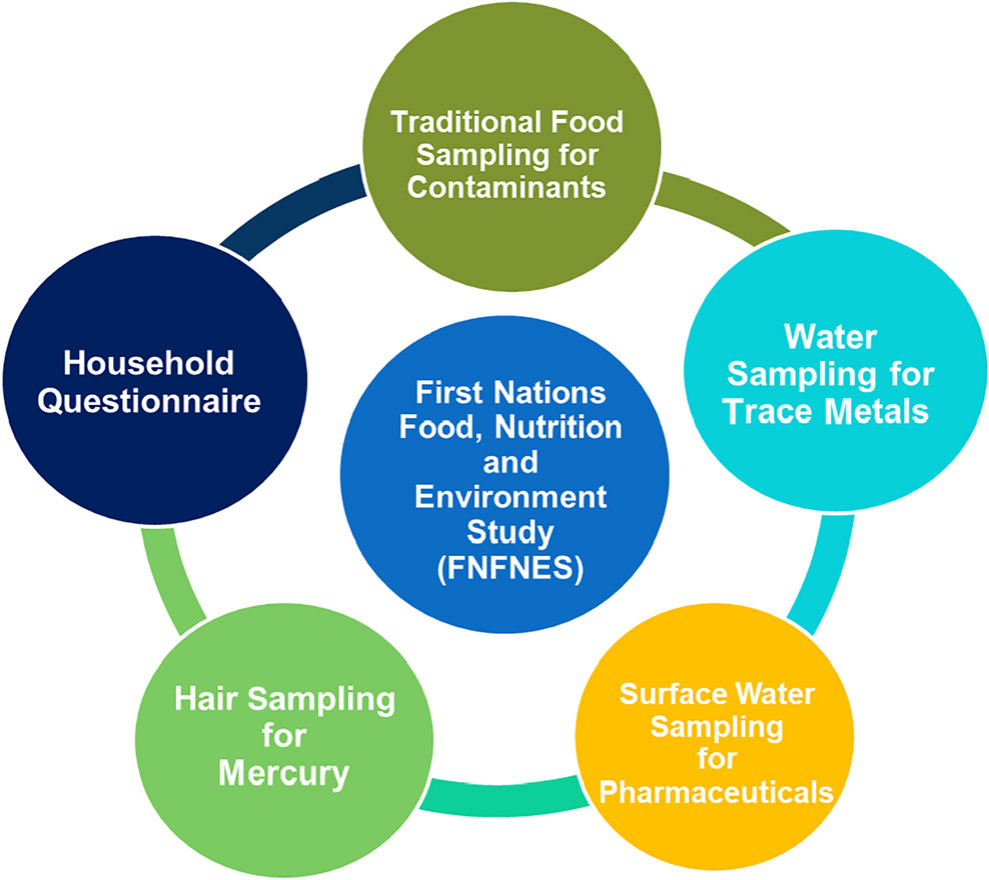
The five research components of FNFNES

The five components of the FNFNES are presented in Fig. 1.

1. Household interviews: each participant was asked a series of questions that focused on foods consumed, health, lifestyle and socio-economic issues, household composition and household food security.
2. Tap water sampling for trace metals: tap water samples were collected from 20 participating households that were representative of the water distribution systems in every community.
3. Surface water sampling for pharmaceuticals: water samples were collected from three separate sites chosen by the participating community to analyze for the presence and amount of agricultural, veterinary and human pharmaceuticals and their metabolites.
4. Hair sampling to estimate mercury exposure: all participants were invited to provide a hair sample.
5. Traditional food sampling for contaminant content: each community identified and collected up to 30 traditional foods that were commonly consumed and that people were concerned about for the analysis of a suite of environmental contaminants and nutrients according to local priorities.

### Vetting of results with communities and regions

**Fig. 2:**
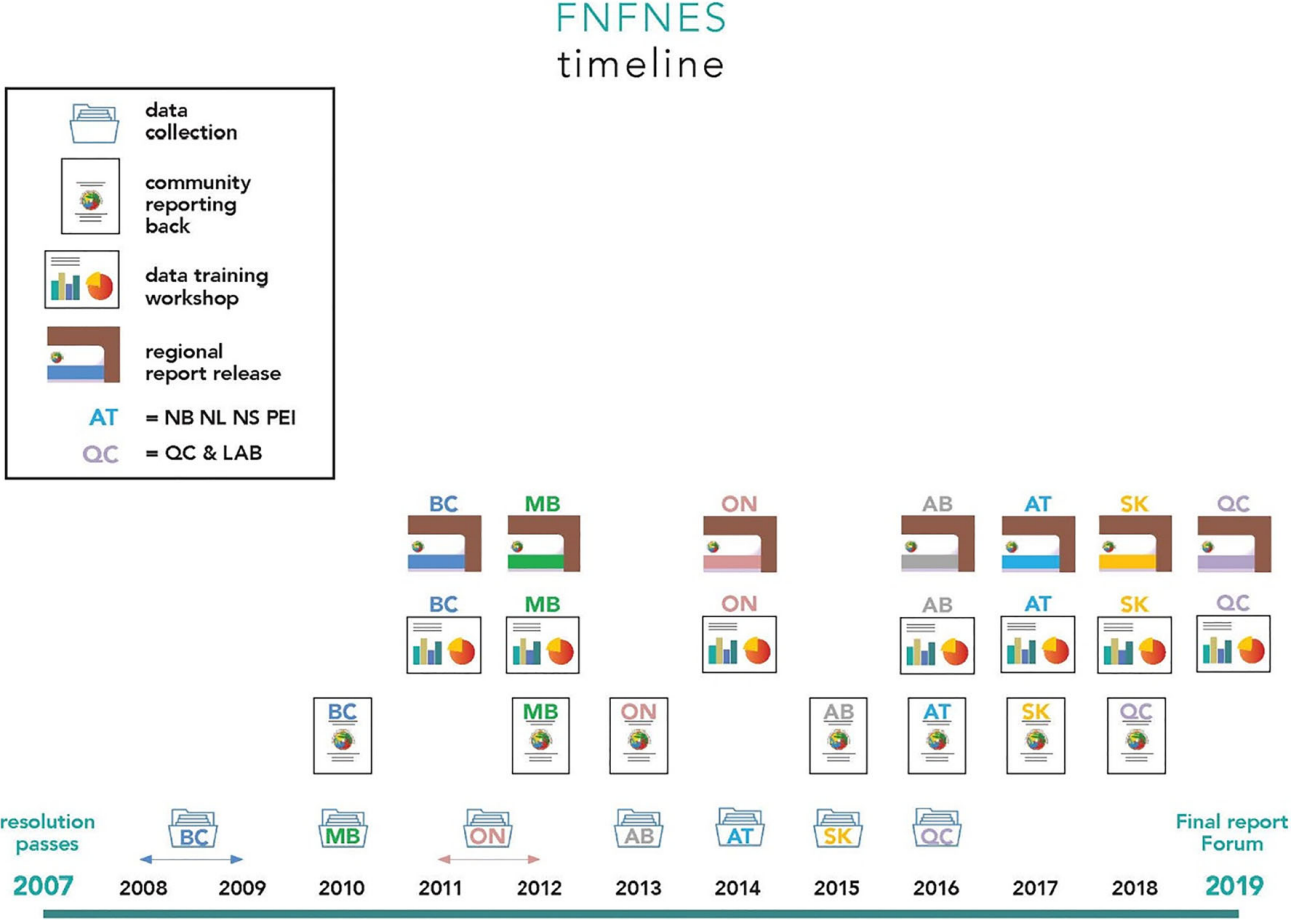
The timeline for data collection, community reporting, data training workshops and release of regional reports in the eight AFN regions

Upon completion of community data analyses, draft reports were submitted to each First Nation partner for the initial review. Verification meetings were undertaken in each community, and feedback was incorporated into the local reports, which were only released to the First Nation. Each First Nation would have the discretion to determine whether or not to share their community-level results. Data training workshops were held where community representatives were trained on how to read and interpret their data, perform basic data analyses, write draft proposals and discuss current funding opportunities available to them. Data training workshops created an environment for representatives to work together, brainstorm, and share success stories and experiences. At the regional level, both the AFN Regional Chief and the regional health authority were consulted on the interpretation of the results before the release of a Regional Report at a regional event, such as an all-Chiefs meeting, with opportunities for the exchange of ideas on the pertinence of the results to First Nations, health implications and policy development. The timeline for data collection, community reporting, data training workshops and release of regional reports in the eight AFN regions is presented in Fig. 2.

## Results

**Fig. 3:**
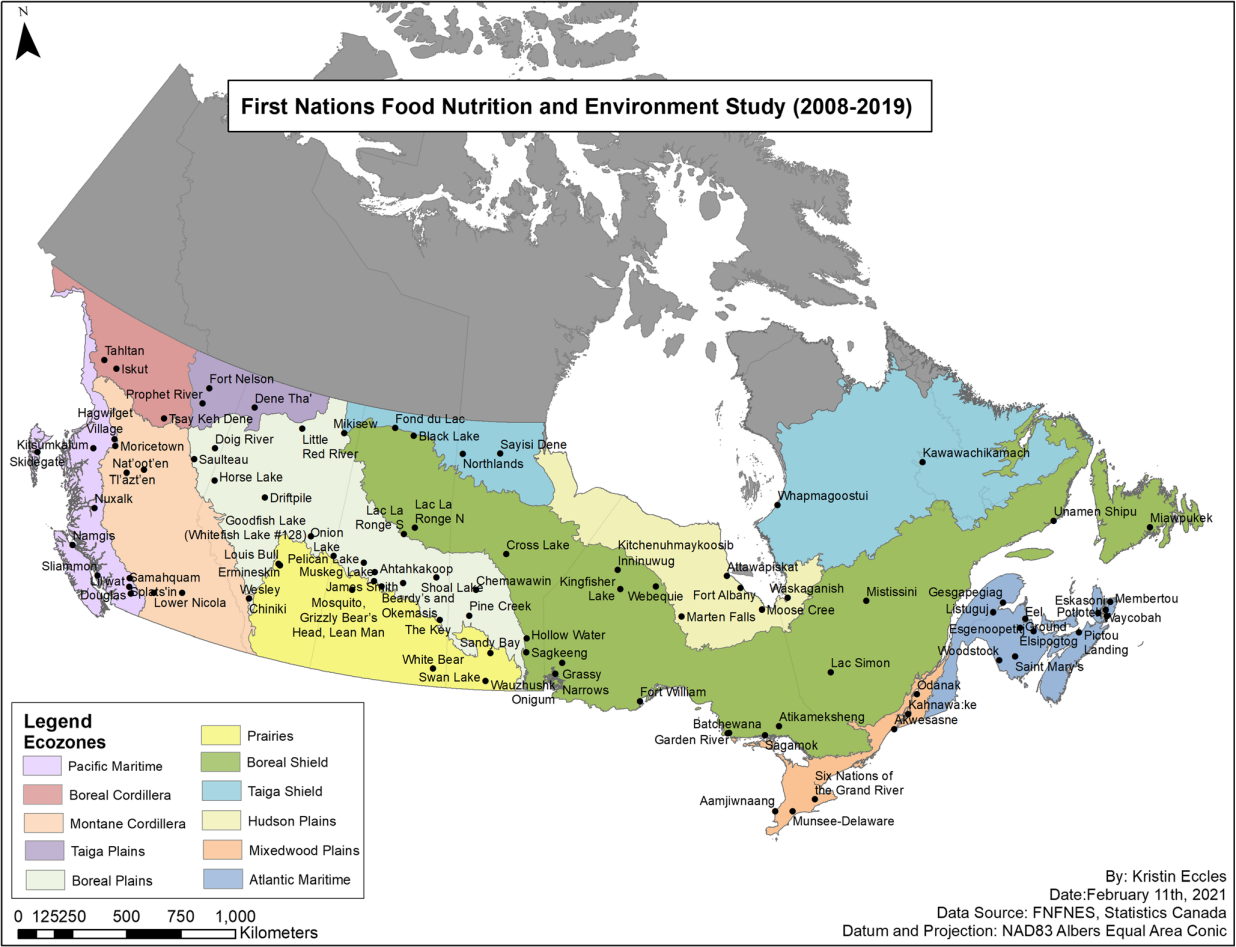
Map of participating communities, AFN regions and ecozones

A summary of the First Nations approached, selected and having participated in the FNFNES in each AFN region is presented in Table 1. Out of the 117 First Nations invited, 22 communities declined to participate after the initial consultation. Eighteen alternate communities were approached, and 17 agreed to participate. Two First Nations selected with certainty did not have an alternate (one community did not have an ecozone alternate, and one community did not have an alternate because of its population size). One purposely selected community chose not to participate. Three First Nations withdrew partway through data collection and were therefore dropped from the analyses for the region. One First Nation in the Saskatchewan AFN region had occupied reserves in two ecozones (Boreal Plains and Boreal Shield); a decision was made to split the First Nation into two sites by an ecozone boundary. A total of 92 First Nations located in 11 ecozones completed the five general study components of FNFNES. The location of First Nations by AFN region and ecozone is presented in Fig. 3. Most ecozones extend over two or more regions, such as the Boreal Plains and Boreal Shield. On the other hand, the AFN BC region includes three ecozones (Pacific Maritime, Boreal Cordillera and Montane Cordillera).

**Table 1:** A summary of the First Nations approached, selected and having participated in the FNFNES in each AFN region

Characteristic	All regions	British Columbia	Alberta	Saskatchewan	Manitoba	Ontario	Quebec-Labrador	Atlantic
Year(s) of data collection	2008 to 2016	2008 and 2009	2013	2015	2010	2011 and 2012	2016	2014
# of First Nations with on-reserve population in 2008	583	198	46	70	63	137	40	31
Population on-reserve (2008)	413,205	58,876	63,707	61,564	78,415	82,952	49,597	18,454
Original sample allocation	92	20	10	12	12	18	9	12
Communities approached	117	23	16	19	12	19	13	15
Selected with certainty due to population/ecozone	9	0	1	1	3	0	2	2
Random selection	82	19	9	11	9	16	8	10
Alternates	18	2	4	6	0	1	2	3
Invited	8	2	2	1	0	2	1	0
Refusals	22	2	6	6	0	1	3	4
Selected with certainty	2	0	0	0	0	0	1	1
Randomly selected	18	2	4	6	0	1	2	3
Alternate	1	0	1	0	0	0	0	0
Invited	1	0	1	0	0	0	0	0
Withdrew during study	3	0	0	0	3	0	0	0
Participating communities	92	21	10	13*	9	18	10	11
Selected with certainty due to population/ecozone	6	0	1	1	3	0	0	1
Randomly selected	62	17	5	5	6	15	7	7
Alternate	17	2	3	6	0	1	2	3
Invited	7	2	1	1	0	2	1	0

*One community randomly selected was split into two separate communities due to the location of communities in two ecozones

  Table 2 presents the participation rate and the characteristics of participants, including household information (age, gender, household size) by region. Overall, a total of 6487 or 78% of adults contacted for this Study completed the household questionnaire component of FNFNES. Although the randomization process ensured that there would be an equal chance of either gender being selected to participate, a higher percentage of females (66%) participated than males (34%). The average age of males and females was similar (44 and 45 years, respectively). Sixty-nine percent of households contained dependents under the age of 18 years, and the average household size across the regions was five people. At the regional level, the average number of people living in households ranged between four and six while the percentages of households with children were 58% in British Columbia, 68% in Alberta, 69% in Saskatchewan, 74% in Manitoba, 48% in Ontario, 55% in Quebec-Labrador, and 48% in the Atlantic region.

**Table 2:** Participation rate and the characteristics of participants, including household information

Characteristic	All regions	British Columbia	Alberta	Saskatchewan	Manitoba	Ontario	Quebec-Labrador	Atlantic
Participation rate of household questionnaire	78%	68%	70%	84%	82%	79%	71%	90%
Number of participants	6487	1103	609	1042	706	1429	573	1025
Females	4277	706	387	721	477	896	420	670
Males	2210	397	222	321	229	533	153	355
Mean age (SE)								
Females	44 (0.5)	45 (1.7)	42 (1.1)	43 (1.3)	43 (1.0)	45 (0.7)	42 (0.4)	43 (0.5)
Males	45 (0.9)	46 (1.7)	42 (3.0)	44 (1.3)	44 (3.5)	46 (1.9)	48 (0.5)	43 (0.8)
Mean household size (SE)	5 (0.1)	4 (0.2)	6 (0.3)	5 (0.3)	6 (0.4)	4 (0.1)	5 (0.3)	4 (0.1)
Percentage of households with children under the age of 18 years	69%	58%	68%	69%	74%	48%	55%	48%

Traditional food systems remain foundational to First Nations. The average daily intake of traditional food was 61 grams, while some adults reported eating more than 1000 grams (Batal et al. 2021a). On days when traditional food was eaten, the intake of almost all nutrients was significantly higher while the intake of saturated fat was lower (Batal et al. 2021a, 2021b). The diet of First Nations adults does not meet nutrition recommendations; intake of vitamins A, D and C, folate, calcium and magnesium are inadequate (Batal et al. 2021c).

First Nations living on-reserve experience extremely high rates of food insecurity: an average of 48% (range between 24% and 60%) of households are food insecure, and the rate is 3 to 5 times higher than the food insecurity rate reported for the general Canadian population (12%) (Batal et al. 2021d). Fish harvest and consumption were found to be important contributors to nutritional health and food security among First Nations (Marushka et al. 2021a).

Traditional food is safe for consumption, with two primary exceptions. The use of lead-based ammunition resulted in very high levels of lead in many harvested mammal and bird samples. As a result, there is an elevated risk of exposure to lead for some adults and for women of childbearing age (Chan et al. 2021). Large predatory fish (such as walleye and northern pike) in some areas have higher levels of mercury, and some women of childbearing age have elevated levels of exposure, particularly in the northern parts of Saskatchewan, Manitoba, Ontario and Quebec (Chan et al. 2021). This is corroborated by the hair mercury monitoring results. While most (95.5%) of the 3404 participants who provided hair samples had mercury levels below the Health Canada mercury guideline of 2 μg/g, women of childbearing age (19–50) and older individuals (51+) living in northern ecozones and Quebec tend to have a higher hair mercury exposure (Tikhonov et al. 2021).

Taste and colour of water are two common reasons that limit the use of drinking water. A total of 453 (30%) of the 1516 households that had drinking water tested showed exceedances for metals that affect taste and colour. Observed exceedances that might pose human health concerns were as follows: 128 or 8.4% for lead, 4.0% for manganese, 1.6% for uranium, 1.3% for aluminum and 0.2% for copper (Schwartz et al. 2021a). Regular maintenance and improvement of the water treatment and/or delivery system needs to be implemented to improve the drinking water supply quality. Pharmaceuticals such as caffeine and 17α-ethinylestradiol were detected in surface waters of some First Nations communities, suggesting potential sewage contamination, but the levels would not pose a threat to human health (Schwartz et al. 2021b).

The health of many First Nations adults is compromised with very high rates of smoking and obesity (i.e., double the obesity rate among Canadians) and with one fifth of the adult population suffering from diabetes (Batal et al. 2021e). Dietary exposure to persistent organic pollutants was found to be associated with rates of type 2 diabetes among First Nations (Marushka et al. 2021b).

## Discussion

This is the most comprehensive statistically representative study on nutrition and the environmental health of First Nations ever completed in Canada. A full picture of diet quality, nutrition and health status, food security, drinking water quality and food safety has been obtained, and this important information can be used by First Nations, risk assessors and policymakers to develop effective programs to promote community health and well-being. An Indigenous worldview recognizes the interconnectedness of all forms of life and the natural environment and holds the earth, *Mother Earth*, as sacred. This is the reason that the information collected by the FNFNES is also important for community empowerment, environmental stewardship and the general promotion of good health by and for First Nations peoples in Canada.

Many invaluable lessons can be learned from the design and implementation of FNFNES. There is a long history of research involving First Nations that has been mired by racist, privileged and colonialist attitudes and has not been respectful and inclusive of Indigenous worldviews (Anderson 2019; Mosby 2013; McCallum 2017). First Nations peoples have reclaimed governance over the research agenda by supporting community-based participatory approaches, which evolved from the early 1990s and led to the establishment of the OCAP® principles, a set of standards that establish how First Nations data should be collected, protected, used and shared (https://fnigc.ca/ocap-training/). They are the de facto standard for how to conduct research with First Nations. Chapter 9 of the Tri-Council Policy Statement: Ethical Conduct for Research Involving Humans (TCPS 2) is designed to serve as a framework for the ethical conduct of research that respects the diverse worldviews of Indigenous Peoples (https://ethics.gc.ca/eng/tcps2-eptc2_2018_chapter9-chapitre9.html). These protocols are in alignment with the United Nations Declaration on the Rights of Indigenous Peoples (UNDRIP) that establishes a universal framework of minimum standards for the survival, dignity and well-being of the Indigenous peoples of the world (United Nations 2007). As a result, the relationship between academic researchers and Indigenous Peoples in Canada has been redefined. A community-based participatory approach requires meaningful partnerships built early on, and with the research mandate coming directly from the people involved. In the case of this Study, we obtained the resolution from the AFN before the start and the endorsement from the regional and/or national First Nations leadership throughout the process. All data were collected by the 542 Community Research Assistants recruited locally by the participating First Nations and trained by the research team. Local experts first vetted the preliminary results, and their input and comments were incorporated into the reports. Data training workshops were conducted to help community members read their community-specific data files and use the results to develop local programs or further studies. Therefore, FNFNES is a good example of how adopting such an approach can lead to success in obtaining data, synthesizing useful knowledge and rebuilding capacity both in local First Nations and at the regional level through partnerships.

With over $12 million in funding support from the First Nations and Inuit Health Branch (FNIHB), formerly of Health Canada and now Indigenous Services Canada, researchers from the University of Ottawa and the Université de Montréal worked in partnership with the AFN, the FNIHB and the participating First Nations to develop and implement FNFNES. This partnership was formally established by the signing of the Community Research Agreement that clearly stated the roles of each partner. At the national level, AFN has full governance of and access to the aggregated data and takes the lead for the approval process for publications and conference presentations, reports, funding opportunities, knowledge translation, and intervention strategies. Individual First Nations continue to possess and control their own data and can ask the research team for assistance in exploring them further as the need arises. The principal investigators were responsible for ensuring the quality of the science, the training of the highly qualified personnel and the publication of the results in reports, peer-reviewed journals and scientific conferences. In total, FNFNES involved 50 nutritionists from across Canada to supervise the data collection and 20 staff members to coordinate the research. FNFNES also provided participatory research training to 40 graduate and undergraduate students.

### Community relevance and flexibility

While many partnerships were forged through FNFNES, the relatively long process and continuous introspection allowed for the development of the community engagement and data collection tools. Further lessons were learned about how to build better research relationships.

Community-based participatory research requires a large investment in social capital—from the first through to the final day—throughout and beyond the scope of the research mandate. The benefits of this include the possibility of more relevant research questions, increased data use and dissemination, and the potential to establish sustainable partnerships for project expansion or future research, all of which can lead to better policy and health outcomes.

The need to be flexible is essential and challenging. In this Study, we worked to strike a balance between strictly adhering to study protocols—important for comparing data between regions and across years—while adapting to meet the distinct needs of each community. It is important to emphasize the need for sufficient community engagement *prior to and throughout* data collection as a precursor for successful outcomes. In First Nations where communication and relationship building was strong, particularly around the potential benefits of the Study, leadership tended to be highly supportive, and a community champion would typically emerge.

### Cultural safety

A successful collaborative partnership has a clear set of standard operating procedures (SOP). The FNFNES team established an SOP that included the development of culturally appropriate protocols and a well-defined series of guiding principles. This enabled us to have well-understood expectations for each party, including different levels of management, coordination of different institutions, and a clear chain of command. We developed and adapted fieldwork protocols that encouraged open communication between partners and safety for all members of the research team. This included study awareness campaigns, training protocols and resources, guidelines for working in remote locations, and check-in procedures. The research team was publicly introduced to the First Nations. This introduction also included a clear description of how long and how often the research team was expected to be in the community and how the information would be shared between the team and the community partners.

### Shortcomings and limitations

The sampling strategy was designed to ensure the findings of this Study are representative at the regional and ecozone level for all First Nations adults living on-reserve south of the 60^th^ parallel, including those who did not participate in the Study. However, every First Nation has unique circumstances (e.g., a local pollution source, lack of accessibility to specific food). The results can serve as baseline reference data and support communities in determining how the results can be used and what kinds of additional complementary local studies may be needed. Therefore, our results should be viewed as baseline information only and further input and validations are needed before applying the results for other First Nations.

The data were collected over a 10-year period. We have adjusted for the temporal changes in population size only. Other potential environmental, demographic or socio-economic changes that may have occurred have not been addressed. This brings some uncertainty to the results described in this series of articles.

The tight timeline of the study design often limited the time and effort that we spent in each community and reduced the Study’s ability to contribute to building capacity in the community much beyond the training of community researchers as interviewers (including 24-hour recalls), sample collectors (hair, tap water, and traditional food) and communications personnel/community liaisons. Moreover, while efforts were made to present results to the First Nation’s leadership and members, some First Nations members were still unaware of the study results after its completion. Early consultation and sustained community engagement are needed, e.g., by identification of community champions and the creation of Community Advisory Circles to help guide and provide knowledge during the data collection period and ensure widespread dissemination of the study findings. Systematic gathering of feedback and continual updating of the methodology are needed to ensure that the Study remains relevant to the needs of the participating First Nations and that its findings are used towards programming.

Some components of the study design may not be culturally appropriate for First Nations. For example, hair sampling for mercury analysis was challenging because, in some First Nations, hair has special spiritual and cultural significance, though traditions and culture vary from nation to nation. This, and other sampling limitations, resulted in only 52.5% (33.4% to 66.5% across the regions) of the respondents to the household surveys providing hair samples (Tikhonov et al. 2021). Alternative methods that are more acceptable may need to be offered, e.g., collection of blood samples. Our knowledge translation efforts were limited to one release of reports to each participating First Nation, one release of each regional report in an appropriate regional gathering, and a national closing workshop held in Ottawa in November 2019. This closing workshop, the First Nations Food, Nutrition and Environment Forum, was attended by 275 delegates from 140 First Nations, including 60 of the participating First Nations. Consistent communication and knowledge exchange channels are required to engage First Nations meaningfully and on a continual basis. Detailed knowledge translation plans are also required to ensure that proper interventions can be implemented following the availability of results for their optimal use at the local, regional and national levels. This implies that appropriate funding is allocated to ongoing engagement and communication activities and that the required partners and funding sources for such activities are identified or even secured at the beginning of the Study.

Many of the analyses conducted for this Study were mainly descriptive and measured at the individual level. Our understanding remains limited about the magnitude of impact from structural determinants such as historical and contemporary political contexts, social structures, and resource distribution (Reading 2015). The structural determinants include factors beyond the control of individuals, including policies, governance and jurisdiction, location, access to appropriate education, housing, and culturally safe health services, as well as social networks on adults’ food and lifestyle. At the individual level, access to resources (money, equipment), knowledge, and a compromised environment have a strong influence on behaviours. Further discussions with representatives from First Nations and Indigenous organizations, such as the efforts spearheaded by the First Nations Food, Nutrition and Environment Forum (November 5–6, 2019), are critical for the contextualization of these results and for the regular appraisal (or monitoring) of the level of commitment from governments and First Nations to addressing the environmental, nutritional and health issues raised by this Study.

## Conclusion

The FNFNES (2008–2018) collected important baseline information for advocacy and future comparisons where First Nations seek to enhance their food sovereignty and governance, as well as environmental stewardship. The results also support the development of future policy and intervention programs at all levels—local, regional and national. Further research is required to elucidate the many associations among the health of the people, their nutrition and food security, and the health of the environment. A major information gap remains in regard to the environmental health and nutrition of First Nations children and youth. Research on the legacy of various government policies and their impacts on the sustainable access to culturally relevant healthy traditional food that is free from contaminants is needed to better drive future policy development (e.g., for improved health outcomes). Community resilience and community-based solutions to human and environmental health issues need to be researched and effective ones shared. We envision research in the future that celebrates diversity, recognizes distinct languages and cultures, promotes individual and community self-determination, builds local capacity, and brings about meaningful positive change for the health of people and the environment.

## Data Availability

Data are owned by each participating community. The Assembly of First Nations is data custodian and any requests will be addressed to AFN through the corresponding author.

## Code availability

Analysis codes can be shared upon reasonable request.
